# X-Ray Crystal Structure of the Ancestral 3-Ketosteroid Receptor–Progesterone–Mifepristone Complex Shows Mifepristone Bound at the Coactivator Binding Interface

**DOI:** 10.1371/journal.pone.0080761

**Published:** 2013-11-19

**Authors:** Jennifer K. Colucci, Eric A. Ortlund

**Affiliations:** Department of Biochemistry and Winship Cancer Institute, Emory University School of Medicine, Atlanta, Georgia, United States of America; Univeristy of California Riverside, United States of America

## Abstract

Steroid receptors are a subfamily of nuclear receptors found throughout all metazoans. They are highly important in the regulation of development, inflammation, and reproduction and their misregulation has been implicated in hormone insensitivity syndromes and cancer. Steroid binding to SRs drives a conformational change in the ligand binding domain that promotes nuclear localization and subsequent interaction with coregulator proteins to affect gene regulation. SRs are important pharmaceutical targets, yet most SR-targeting drugs have off-target pharmacology leading to unwanted side effects. A better understanding of the structural mechanisms dictating ligand specificity and the evolution of the forces that created the SR-hormone pairs will enable the design of better pharmaceutical ligands. In order to investigate this relationship, we attempted to crystallize the ancestral 3-ketosteroid receptor (ancSR2) with mifepristone, a SR antagonist. Here, we present the x-ray crystal structure of the ancestral 3-keto steroid receptor (ancSR2)-progesterone complex at a resolution of 2.05 Å. This improves upon our previously reported structure of the ancSR2-progesterone complex, permitting unambiguous assignment of the ligand conformation within the binding pocket. Surprisingly, we find mifepristone, fortuitously docked at the protein surface, poised to interfere with coregulator binding. Recent attention has been given to generating pharmaceuticals that block the coregulator binding site in order to obstruct coregulator binding and achieve tissue-specific SR regulation independent of hormone binding. Mifepristone’s interaction with the coactivator cleft of this SR suggests that it may be a useful molecular scaffold for further coactivator binding inhibitor development.

## Introduction

Steroid hormones play a crucial role in all but the most basic metazoans, orchestrating the cell-cell communication required to coordinate development, growth, metabolism, immunity and more [1]. These hormones are small lipophilic molecules that act directly on a class of transcription factors termed steroid hormone receptors to mediate their down stream effects. Misregulation of steroid signaling leads to metabolic, immune, and neoplastic diseases. Thus, the steroid receptors (SRs), consisting of the estrogen receptor (ER), progesterone receptor (PR), androgen receptor (AR), mineralocorticoid receptor (MR), and glucocorticoid receptor (GR), are highly targeted for therapeutic intervention.

SRs have a modular domain architecture consisting of a highly variable N-terminal domain, a DNA-binding domain (DBD), a short hinge region, and a ligand binding domain (LBD) [2]. ApoSRs are sequestered in the cytoplasm by heat shock proteins (HSPs), which hold them in a ligand-ready state; they are activated when a steroid hormone binds the ligand binding pocket, remodeling the HSP complex and triggering nuclear import [2,3]. Agonist binding drives a conformational change, whereby helices 3, 4, and 12 (H3, H4, H12, respectively) create a docking surface for coregulatory proteins termed the activation function surface (AF-H) [4,5]. Antagonist binding on the other hand prevents proper packing of H12 against H3 and H4 favoring corepressor interaction. Mutations within the AF-H can disrupt coregulator interaction causing ligand insensitivity [6-8]. Coactivators, interact with SRs via helical LXXLL (L—leucine, X—any amino acid) motifs, and act as intermediaries to RNA Polymerase II and other transcriptional machinery [4,5]. The recruitment of any given coregulatory protein exhibits both ligand- and tissue-specificity dictated by the available coregulator pools and state of the cell [9]. Coregulatory proteins act as conduits to all further transcriptional activation or repression, thus their regulation remains a highly desirable pharmaceutical target. 

Recent efforts to achieve tissue-specific SR-mediated regulation has been focused on developing compounds to block the SR-coactivator interface to modulate certain SR-mediated gene activity. These small molecules, dubbed Coactivator Binding Inhibitors (CBIs), are effective at competing for coactivator binding space and altering downstream transcription [10]. These compounds typically contain heterocyclic cores and possess substituents that mimic the three trussing leucine residues of coactivator proteins [10]. 

 In the absence of HSPs, SRs are inherently unstable, complicating efforts to identify the mechanisms driving ligand specificity and our ability to build robust structure-function relationships. Recent studies have utilized ancestral steroid receptors (ancSRs) to identify the molecular mechanisms that dictated the evolution of ligands specificity among SRs [11-13]. AncSRs display a greater tolerance to mutation while preserving faithful ligand specificity and activation in cells [14,15]. These qualities make ancSRs useful tools to study the selectivity and mechanisms of action of SR-targeting pharmaceuticals [16]. 

 Here we report the structure of the ancestral 3-ketosteroid receptor, ancestral steroid receptor 2 (ancSR2), the ancestor of the AR, PR, MR, and GR. This structure shows the ancSR2—progesterone complex with the SR antagonist mifepristone bound at two surface sites [17]. This structure (2.05 Å) improves the resolution of a previously published ancSR2—progesterone complex (2.75 Å) [17]. Surprisingly, one of the bound mifepristone molecules occupies the coactivator binding space, suggesting a potential use of this drug as a molecular framework for further CBI development. A second bound mifepristone molecule sits at the base of the receptor and interacts with crystallographic symmetry mates; this molecule alters the crystal packing conditions from a previously published structure of the ancSR2—progesterone complex (PDB accession code: 4FN9) [17]. 

## Materials and Methods

### Reagents

Chemicals were purchased from Sigma (St. Louis, MO) or Fisher (Hampton, NH). Mifepristone was purchased from Tocris Biosciences (Bristol, UK). Progesterone was purchased from MP Biomedicals (Santa Ana, CA). The vector for His tagged TEV was a gift from David Waugh (National Cancer Institute). The pLIC_MBP vector was a gift from John Sondek (UNC, Chapel Hill). The ancSR2 LBD was resurrected using well-established protocols and was kindly provided by Dr. Joseph Thornton (University of Oregon, OR) [17]. 

### Expression and purification

AncSR2 LBD was expressed as a 6xHis-MBP fusion protein in BL21(DE3) *E. coli*. Cultures (1.0 L in TB) were grown to an OD_600_ of 0.8 and induced with a final concentration of 400 μM IPTG and 50 μM progesterone at 30 °C for 4 hours. Ancestral SRs, like the extant receptors are inherently unstable in the absence of ligand and adding ligand at induction is required to for soluble overexpression of the recombinant protein. Cell mass was collected by centrifugation at 4 krpm for 20 minutes, resuspended in 150 mM NaCl, 20 mM Tris HCl (pH 7.4), 5 % glycerol, 25 mM imidazole, 0.1 % PMSF and lysed using sonication on ice. The 6xHis-MBP-ancSR2-MBP was initially purified using Ni^2+^ affinity chromatography (HisTrap column, GE Healthcare). Fractions containing ancSR2-MBP were identified by denaturing polyacrylamide gel electrophoresis (SDS-PAGE), pooled and dialyzed against 150 mM NaCl, 20 mM Tris HCl (pH 7.4), 5 % glycerol and 1 mg TEV. Following TEV cleavage, the 6xHis-tagged MBP was removed by an additional Ni^2+^ affinity column. The flow though containing untagged ancSR2 LBD was concentrated using an Amicon Ultra 10K centrifugal filter device (Millipore), concentrated to 3-5 mg ml^-1^, and dialyzed against 150 mM sodium chloride, 20 mM Tris HCl (pH 7.4), and 5 % glycerol. The final purity of the ancSR2 LBD was assessed using SDS-PAGE. In an attempt to exchange progesterone for mifepristone in the ligand binding pocket (LBP), mifepristone (50 μM; approximately 500-fold molar excess) was added to the ancSR2-progesterone complex for 30 minutes at 4 °C and then centrifuged at 14 K rpm for 1 minute to clarify the solution prior to crystallization trails.

### Crystallization, data collection, structure determination and refinement

**Figure 1:**
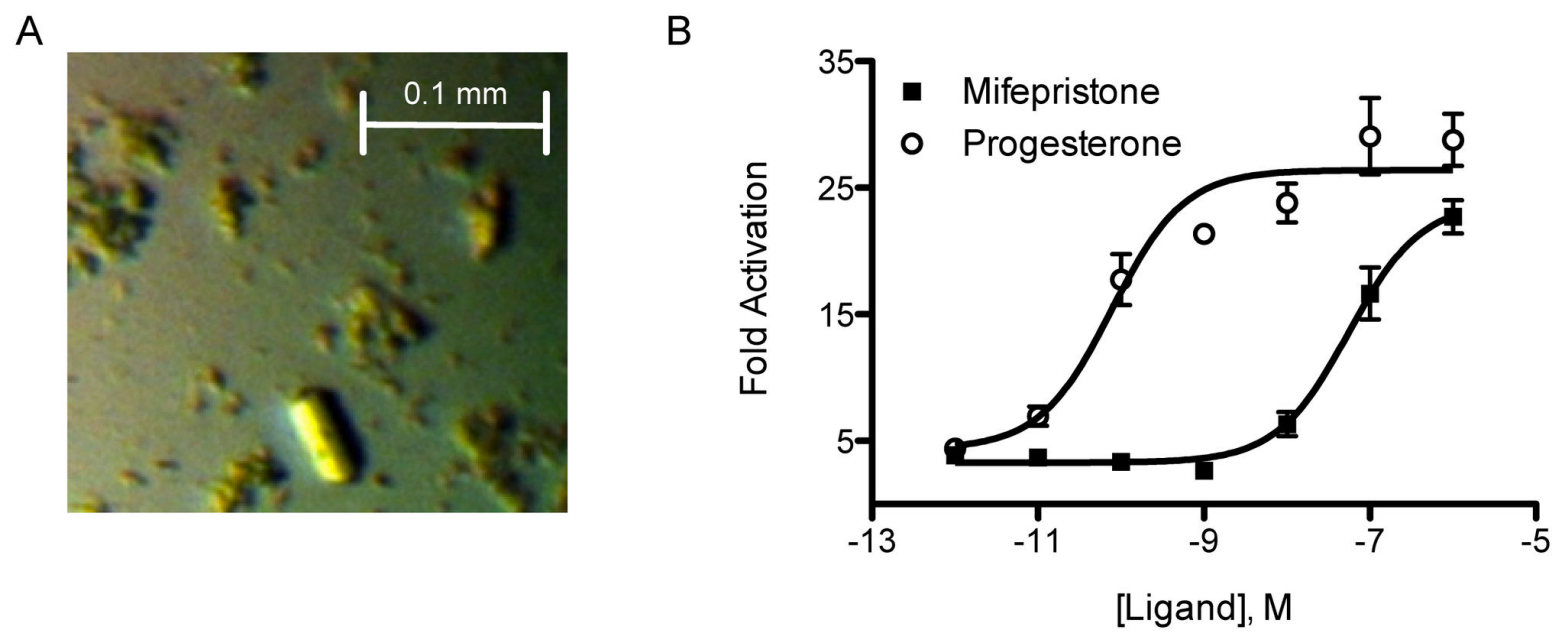
Crystals of the ancSR2–progesterone–mifepristone complex and *in* *vitro* activation data. A. The ancSR2–progesterone–mifepristone ternary complex crystals measured approximately 50 x 20 x 20 microns. B. In luciferase reporter assays, ancSR2 is strongly activated by mifepristone (EC_50_ = 56 ± 1.2 nM) as well as progesterone (EC_50_ = 78 ± 1.7 pM).

Orthorhombic crystals of the ternary ancSR2 LBD–progesterone–mifepristone complex were grown by hanging drop vapor diffusion at 22 °C from solutions containing 1.0 μL of protein at 3-5 mg mL^-1^ protein and 1.0 μL of the following crystallant 0.8 M MgSO_4_, 10 % glycerol, 0.1 M MES (pH 6.0) (Figure 1A). Crystals of the ancSR2 complex grew in P2_1_2_1_2_1_ space group with one monomer in the asymmetric unit. Crystals were cryoprotected by transient soaking in crystallant containing 20 % glycerol and were flash-cooled in liquid N_2_ at 100 K. Data to 2.05 Å resolution were collected at the South East Regional Collaborative Access Team (SER-CAT) 22-BM at the Advanced Photon Source (APS) at Argonne National Laboratory in Chicago, IL using a wavelength of 0.97 Å. Data were processed and scaled with HKL2000 (Table 1) [18]. Initial phases were determined using the previously published ancSR2–progesterone complex (PBD accession code: 4FN9) as the initial search model in Phenix-MR v1.7.1 [19,20]. Residues 2 through 248 were modeled and R_factors_ for the final model are 17.9% and 21.2% for R_work_ and R_free_ respectively. MolProbity was used for model validation, indicating that 98.8% of the residues fall in the most favored regions of the Ramachandran plot with none in disallowed regions [21,22]. The overall MolProbity score was 1.64, placing the structure in the 100^th^ percentile for overall geometric quality among protein crystal structures of comparable resolution [21,22].

**Table 1:** Data Collection and Refinement Statistics.

	ancSR2–Progesterone–Mifepristone
Data Collection	
Space group	P2_1_2_1_2_1_
Cell dimensions	
*a*, *b*, *c* (Å)	53.9, 75.0, 95.7
*α*, *β*, *γ* (°)	90.0, 90.0, 90.0
Resolution (Å)	2.05 (2.12 - 2.05)**^*a*^**
R_merge_ (%)	19.8 (81.3)
I/ σ	11.8 (3.9)
Completeness (%)	99.4 (95.9)
Redundancy	7.5 (5.3)
Refinement	
Resolution (Å)	2.05
No. reflections	187,780
R_work_/ R_free_ (%)	17.9/21.2
No. atoms	
Protein	2,032
Ligand	103
Water	153
B-factors	
Protein	30.7
Ligand	41.2
Water	38.5
R.m.s deviations	
Bond lengths (Å)	0.013
Bond angles (°)	1.7
Ramachandran Outliers	3
Molprobity Score	1.64

*a* Values in parentheses are for highest-resolution shell.

### Reporter gene assays

AncSR2 LBD, was cloned into the Gal4-DBD-pSG5 vector; 31 amino acids of the glucocorticoid receptor hinge containing the nuclear localization signal-1 were inserted between the DBD and the LBD to ensure nuclear localization and conformational independence of the two domains [17]. The hormone-dependent transcriptional activity of resurrected ancestral receptors and their variants was assayed using a luciferase reporter system. CHO-K1 cells were grown in 96-well plates and transfected with 1.0 ng of receptor plasmid, 100 ng of a UAS-driven firefly luciferase reporter (pFRluc), and 0.1 ng of the constitutive phRLtk Renilla luciferase reporter plasmid, using Lipofectamine and Plus Reagent in OPTIMEM (Invitrogen). After 4 hours, transfection medium was replaced with phenol-red-free αMEM supplemented with 10% dextran-charcoal stripped FBS (Hyclone). After overnight recovery, cells were incubated in triplicate with the hormone of interest from 10^-12^ to 10^-6^ M for 24 hours, then assayed using Dual-Glo luciferase (Promega). Firefly luciferase activity was normalized by *Renilla* luciferase activity. Luminescence was read using a Synergy 4 microplate reader (BioTek). Dose-response relationships were estimated using nonlinear regression in Prism4 software (GraphPad Software, Inc.); fold increase in activation was calculated relative to vehicle-only (DMSO) control.

## Results

### Overall structure

Mifepristone is a highly potent SR antagonist, with strong antiprogestagen, antiglucocorticoid, and antiandrogen properties [23,24]. Clinically, it is used as an abortifacient, emergency contraceptive, and as treatment for Cushing’s Syndrome [23,25]. Mifepristone has been shown to bind SRs within their LBP, leading to the recruitment of corepressor proteins [26] or the stabilization of the SR-heat shock protein 90 (HSP90) complex [27]. To gain insight into mechanism by which mifepristone represses 3-keto SRs, we tested mifepristone’s effect on ancSR2-driven gene expression via a luciferase reporter assay. Surprisingly, mifepristone activates ancSR2 with an EC_50_ of 56 nM (Figure 1B). Although this is several orders of magnitude lesspotent than progesterone activation, the EC_50_ is on par with the EC_50_ value for other SR-targeting pharmaceuticals. This weak or partial agonism has been observed for GR in certain cell types and at high enough receptor concentration [28,29]. Thus, mifepristone agonism may be a relic ancSR2. To gain insight into how mifepristone specifically activates ancSR2 while repressing all modern 3-keto SRs, we attempted generate a structure of the ancSR2-mifepristone complex via ligand exchange.

**Figure 2:**
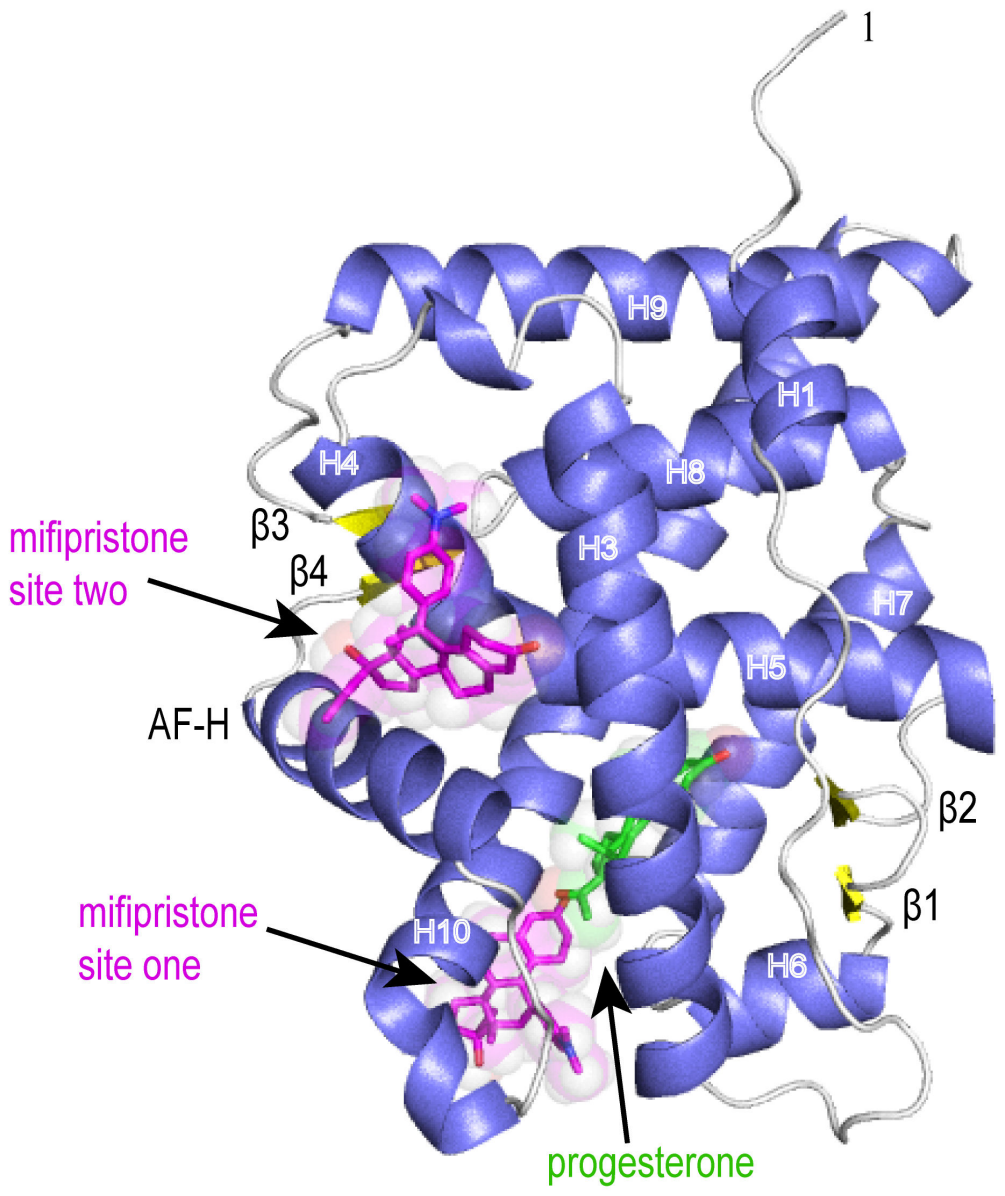
Overall structure of the ancSR2–progesterone–mifepristone complex. Overall structure of the ancSR2 LBD with bound progesterone and mifepristone shown as green and magenta, respectively with oxygens, colored red. Helices are blue, β-sheets are yellow, loops are white. Figures were generated in PyMol (Schödenger, LLC).

 The crystal structure of the ancSR2–progesterone–mifepristone complex (PDB accession number 4LTW) shows that the receptor maintains the canonical steroid receptor fold consisting of a three-layered alpha-helical bundle with four beta strands (Figure 2). Our previously published lower resolution structure of the ancSR2—progesterone complex contained two ancSR2 monomers in the asymmetric unit within the P2_1_2_1_2_1_ space group [17]. Despite highly similar crystallization conditions, addition of mifepristone altered crystal packing and reduced the number of monomers within the asymmetric unit from two to one. 

### Mifepristone binds at two distinct surface sites

**Figure 3:**
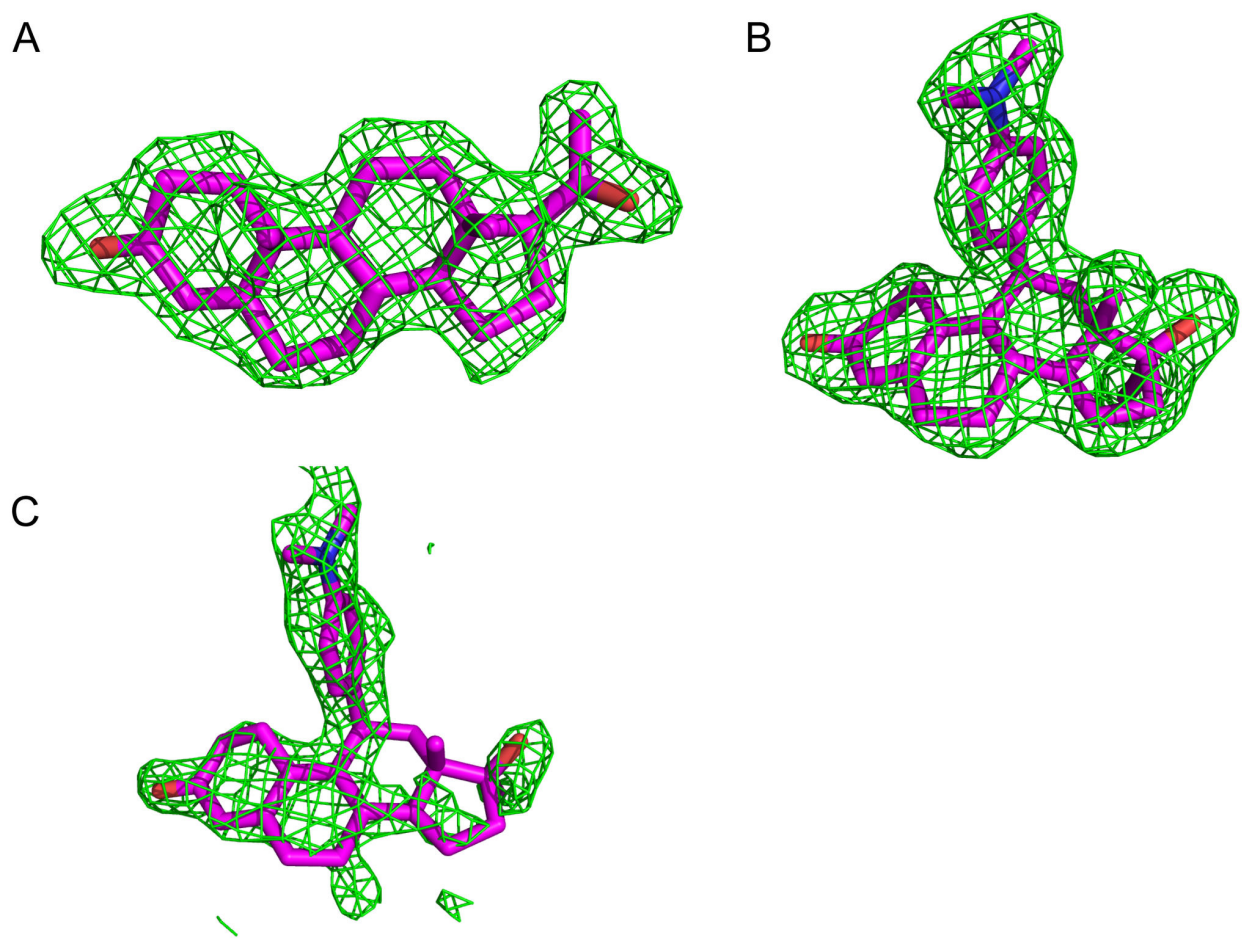
Omit maps of bound ligands. F_o_-F_c_ electron density (green) contoured to 2 σ showing evidence for bound ligand. Omit maps were generated by removal of the ligand from the structure and running 3 cycles of gradient energy minimization and B-factor optimization in PHENIX (version dev-1423) to minimize model bias. A. Electron density within the LBP corresponding to the volume of progesterone. B. Electron density at the base of the receptor corresponding to the volume of mifepristone (site-one). C. Electron density at the coactivator cleft corresponding to the volume of mifepristone (site-two).

**Figure 4:**
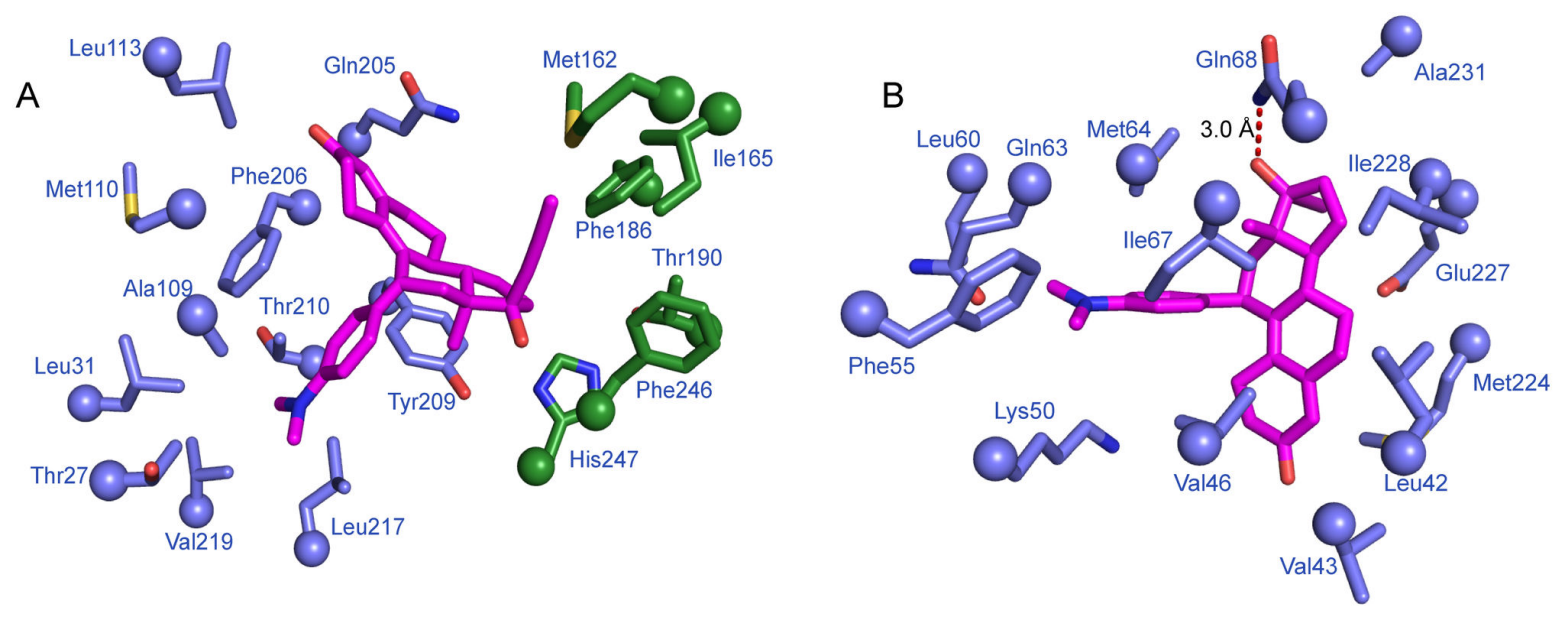
Mifepristone binding site interactions. ancSR2 is shown in slate blue; mifepristone is shown in magenta (oxygens, red; nitrogens, blue). Residues within 4.2 Å of the ligand are shown. A. Site-one mifepristone interacts with both the monomer in the asymmetric unit as well as residues in a symmetry mate (forest green). B. Site-two mifepristone interacts with residues in the ancSR2 coactivator cleft.

Surprisingly, F_o_-F_c_ omit electron density shows clear evidence for the presence of progesterone within the LBP (Figure 3) suggesting that mifepristone failed to exchange with this steroid hormone *in vitro* despite being in nearly 500-fold molar excess. However, initial F_o_-F_c_ electron density clearly showed two well-ordered mifepristone molecules located at distinct surface sites on the receptor which we refer to as “site-one” and “site-two” (Figure 3). Site-one mifepristone makes extensive hydrophobic contacts along helices 3, 7, and 10 of the monomer within the asymmetric unit (AU) and with helices 9 and 10 and the C-terminus of a crystallographic symmetry mate (Figure 4). Site-one mifepristone buries a total surface area of 413.5 Å^2^ between both the monomer located in the AU and the crystallographic symmetry mate [30]. Analyses using the Proteins, Interfaces, Structures, and Assemblies (PISA) server shows a complex significance score of 0.00, suggesting that this interaction plays a role in crystal packing but is not biologically significant [30].

**Figure 5:**
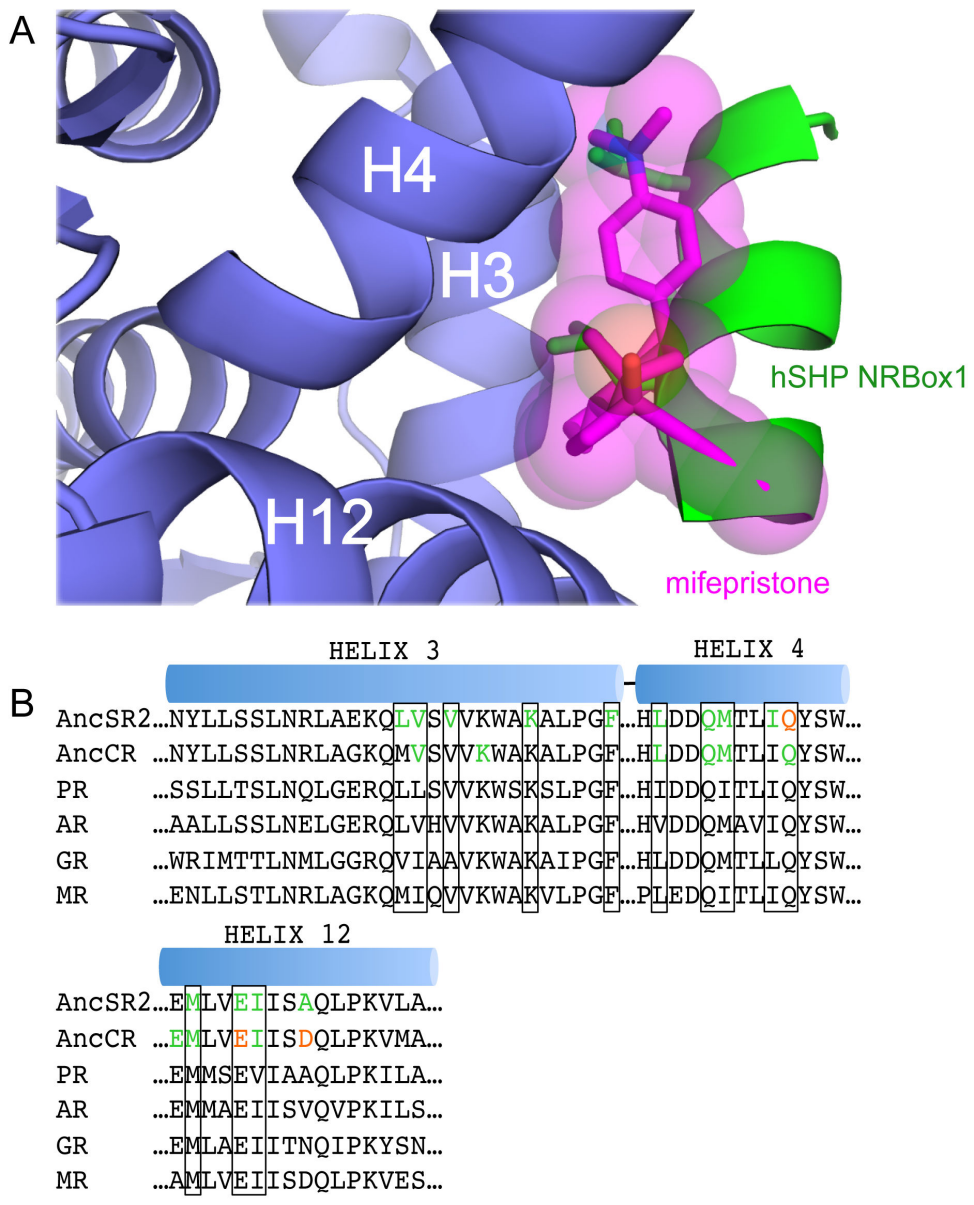
Mifepristone occupies the coactivator protein space. A. ancSR2–progesterone–mifepristone (protein, slate blue; mifepristone, magenta) was overlaid with ancestral corticoid receptor–deoxycorticosterone–hSHP NRBox1 (PDB accession code: 2Q3Y) complex. Mifepristone occupies the same space as the hSHP NRBox1 peptide (green, leucine side chains shown as sticks). B. *Sequence alignment of ancestral and extant 3-ketosteroid receptor coactivator binding clefts*. Sequence alignments of the AncSR2, AncCR, Progesterone Receptor (PR), Androgen Receptor (AR), Glucocorticoid Receptor (GR), and Mineralocorticoid Receptor (MR) coactivator binding clefts. Hydrophobic interactions (green) and hydrogen bonds (red) are shown for the interaction between AncSR2–mifepristone site-two and AncCR–SHP. Conserved residues across the SR lineage are indicated by a black box.

 Surprisingly, a second surface mifepristone was bound to the interface of helices 3, 4, and 12, which is used to recruit coregulator proteins to drive transcriptional activation. Superposition with the ancestral corticoid receptor (ancCR)–deoxycorticosterone–small heterodimer partner (SHP; NR0B2) NRBox1 peptide complex (PDB accession code 2Q3Y), the most closely-related SR, reveals that mifepristone occupies the same position as a coregulator peptide (Figure 5A) [13]. Thus, site-two mifepristone would compete with a coactivator for binding to the coactivator cleft. Site-two mifepristone is coordinated by extensive hydrophobic interactions at the coactivator cleft with an interface surface area of 402.6 Å^2^ [30]. Twelve of the thirteen residues making these hydrophobic contacts are conserved across 3-ketoseteroid receptors (Figure 5B). The C17-hydroxyl group of mifepristone makes a hydrogen bond with the amine group of the conserved Gln68 (Figures 4B, 5B). 

 Unlike site-one mifepristone, site-two mifepristone does not make any crystal contacts, suggesting its binding may be biologically significant. However, there is a very low predicted free energy of binding (1.4 kcal/mol) between the ligand and receptor, indicating low affinity binding [30]. Further, site-two mifepristone was found to have a refined occupancy of 0.84, indicating that the receptor is not fully saturated with mifepristone at the coactivator cleft, despite a final concentration of approximately 25 μM in the crystallization drop. 

### Improved resolution of the ancSR2-progesterone structure permits visualization of D-ring contacts

**Figure 6:**
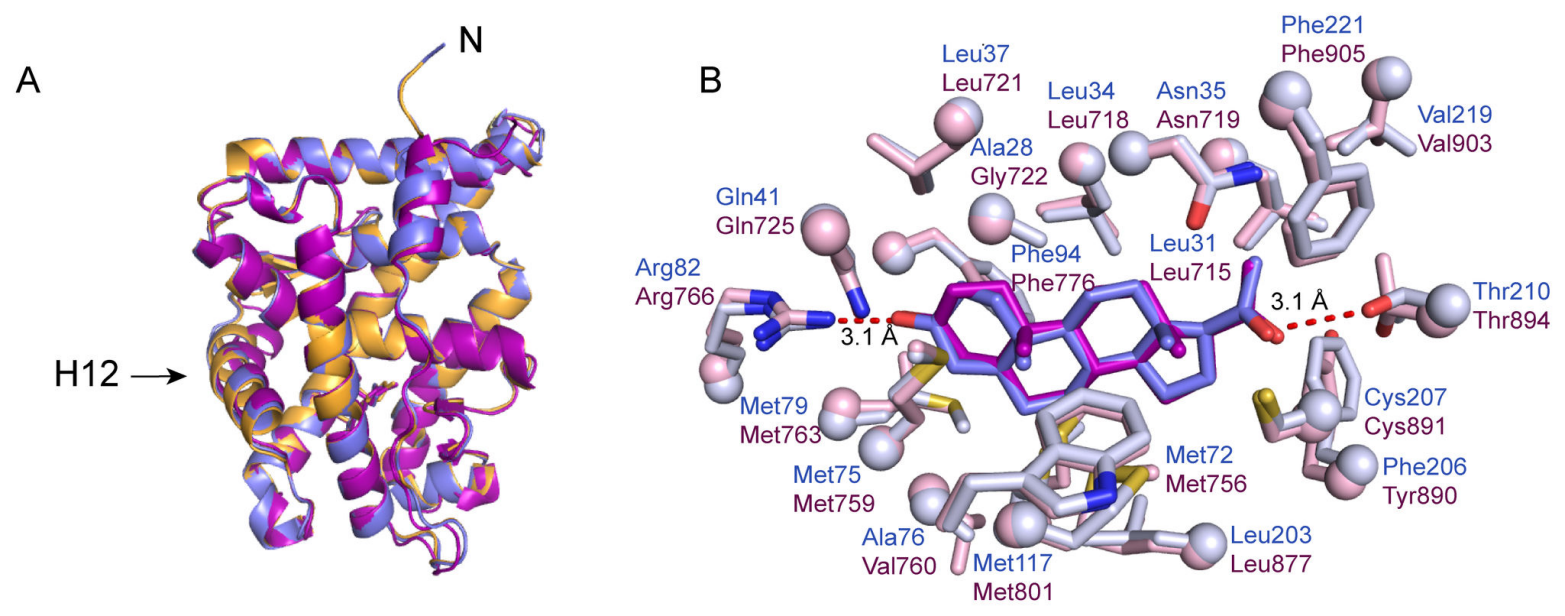
Global alignment of progesterone-bound steroid receptors. A cartoon representation of the ancSR2–progesterone–mifepristone (slate blue), ancSR2–progesterone (orange, PDB accession code: 4FN9), and progesterone receptor–progesterone (purple, PDB accession code 1A28) complexes overlay with high overall structural similarity. B. *Ligand adopts an identical position and conformation in two progesterone-bound receptor structures*. The ancSR2–progesterone–mifepristone (ligand – slate blue, receptor – light blue) and progesterone receptor–progesterone (ligand – magenta, receptor – light pink, PDB accession code 1A28) structures show identical positioning and conformation of the ligand within the ligand binding pocket. Progesterone makes hydrogen bonds with Arg82 and Thr210 in both structures (dashed red lines).

The structure overlays very closely with the structures of both the ancSR2–progesterone complex (PDB accession code: 4FN9) and the PR–progesterone complex (PDB accession code: 1A28); the root mean squared deviation values for all atoms between these structures are 0.3 Å and 0.6 Å, respectively (Figure 6A). Progesterone sits within the LBP, adopting an identical position and orientation as the ligand within the PR-progesterone complex structure (PDB accession code: 1A28) (Figure 6A). Progesterone makes extensive hydrophobic interactions with LBP in addition to two key hydrogen bonds. The first is between glutamate 41 and the 3-keto group of progesterone. It is this interaction that allows ancSR2 to differentiate between estrogen-like compounds and progestagens, androgens, and corticosteroids. Finally, the increased resolution structure allowed for unambiguous modeling of the progesterone’s C20 carbonyl, which is a critical moiety dictating ligand binding and receptor activation. 

## Discussion

This structure is higher resolution than the previously published 2.75 Å ancSR2–progesterone structure [17]. Proper assignment of the hydrogen bond network guiding ligand binding to the LBP is absolutely critical to understand the conserved mechanism of activation across all 3-ketosteroid receptors. In the previous ancSR2–progesterone complex, the orientation of progesterone’s C20 carbonyl was ambiguous and two equally probable H-bonding interactions were possible with asparagine 35 and threonine 210. A central question in the evolution of steroid hormone specificity is whether the allosteric networks that drive modern SR activation were present in the ancestral state or derived in modern proteins. Since the previous structure was not at sufficient resolution to orient the C20 group, we relied on molecular dynamics simulations to guide the final modeling of C20 [11]. Our high-resolution structure reveals that indeed C20 is indeed oriented to accept a H-bond from threonine 210. This is consistent with molecular dynamics simulations of the ancSR2-progesterone complex and crystal structures of the progesterone receptor–progesterone, ancCR–deoxycorticosterone, ancestral glucocorticoid receptor 1–deoxycorticosterone, ancestral glucocorticoid receptor 2–dexamethasone, and mineralocorticoid receptor–aldosterone complexes (PDB accession codes 1A28, 2Q3Y, 3RY9, 3GN8, and 2AA2 respectively) [11-13,31-33]. Resolving the orientation of the ligand C20 confirms that the mechanism of activation among all 3-keto SRs originated in ancSR2 over 500 million years ago.

Attempts to determine the crystal structure of the ancSR2–mifepristone complex via ligand exchanged resulted instead in a crystal structure of ancSR2–progesterone with mifepristone bound at two surface sites. While the inability of mifepristone to exchange for progesterone was unexpected, it prompted us to examine the ability of ancSR2 to bind ligands and coregulators *in vitro*. Despite ancSR2’s ability to respond to a wide array of ligands in mammalian cells [11,17], attempts to measure ancSR2 ligand *in vitro* were not successful, even upon the addition of recombinant HSP90 (data not shown). It is possible that the full HSP90-HSP70-p23-FKBP52-p60 complex is required for ligand binding or exchange *in vitro* [34]. This inability to exchange ligands *in vitro* appears unique to ancSR2 since other ancestral SRs, including ancCR, ancGR1, and ancGR2, as well as the modern SRs are ligand exchangeable [16]. AncSR2 represents the oldest 3-keto SR resurrected thus far; therefore, it is possible that an issue inherent to the reconstruction of the receptor has led to an extremely slow K_off_ preventing *in vitro* ligand exchange. It is well known that SRs display a narrow thermal window of activity and that their active *versus* inactive states are dictated by subtle thermodynamic changes. This is a consequence of both natural selection and neutral drift permitting the fine balance needed to allow the relatively small energetics of hormone binding to drive allosteric changes within the protein to propagate a signal. While too little stability prevents protein folding, too much stability may drive constitutive activation or prevent a dynamic response to ligand. It is possible that the ancSR2 is overstabilized (i.e. samples the active conformation too frequently when complexed to a ligand). This would explain its activation in the presence of mifepristone and its inability to exchange ligand *in vitro*. In line with these observations, ancSR2 is also unable to bind to coregulator peptides *in vitro*. It is well known that there is allosteric communication between the ligand binding sites and coregulator biding site [2,35]. Despite multiple attempts, we were unable to detect coregulator peptide binding to the ancSR2-progesterone complex by fluorescence polarization, while peptide binding to younger SR ancestors (i.e. ancGR1, ancGR2) is robust [16]. Thus, ancSR2 is not able adjust its conformation/ dynamics to accommodate interaction with isolated peptides despite the fact that the structure is superimposable with closely related ancestral SR-ligand-coregulator peptide complexes [12]. 

Given the inability of ancSR2 to bind coactivator or ligand *in vitro*, it was surprising to see a weak secondary mifepristone interaction site at the AF-H surface. It is unclear whether this site is physiologically relevant *in vivo* however, the binding of mifepristone to the coactivator cleft in the crystal structure suggests there may be potential to use mifepristone as a scaffold for designing coactivator binding inhibitors. These novel pharmaceuticals would be instrumental in the treatment of a range of diseases. Currently, there is a struggle to design effective peptidomimetic CBIs due to their inability to permeate the cell membranes [36]. Mifepristone is already well established as having effective extracellular to intracellular transport and thus shows strong scaffold potential. Further work is needed to determine whether mifepristone or mifepristone derivatives are able to compete for the coactivator cleft in extant steroid receptors. 

**Figure 7:**
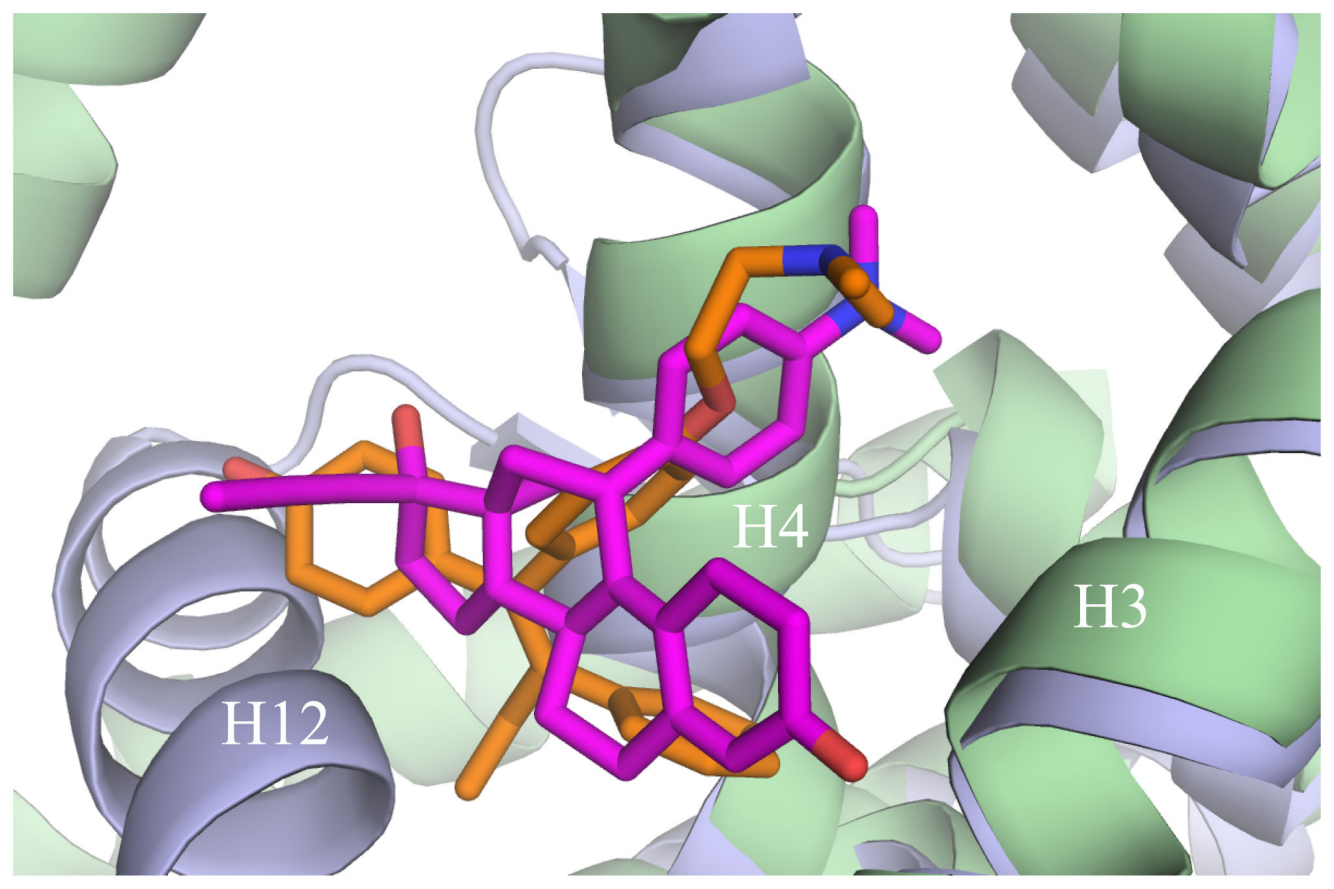
Mifepristone and 4-hydroxytamoxifen show similar binding modes to the steroid receptor coactivator binding cleft. Alignment of the ancSR2–progesterone–mifepristone crystal structure and the ERβ–tamoxifen structure shows both mifepristone and 4-hydroxytamoxifen bound to the coactivator cleft. ancSR2 – slate blue, mifepristone – magenta, ERβ – light green, tamoxifen – orange.

This structure is only the second to show a small molecule bound to the coactivator cleft of a steroid receptor. The first showed the anti-cancer drug 4-hydroxytamoxifen (HT) bound at the coactivator cleft of estrogen receptor beta (ERβ) (PDB accession code 2FSZ) [37]. Overlaying these two structures reveals that the ligands adopt nearly identical positions at the coactivator cleft (Figure 7). Both insert phenolic substituents into the H3/H4 gap and are held in place primarily by hydrophobic interactions. Unlike the ERβ-HT interaction, in which HT doesn’t contact H12 residues, mifepristone makes van der Waals contacts with H12 residues Met224, Glu227 and Ile228 (Figure 4). Together, these structures suggest that small hydrophobic molecules with perpendicular phenolic substituents are prime candidates for CBI development. 

Our present structure suggests that this may extend to small molecules targeting the coactivator cleft binders. The AF-H surface presented in ancSR2 may represent a less dynamic, low energy target for such molecules. Future work should be devoted to investigating whether mifepristone shows potential for acting as a scaffold for further CBI development. Adaptations of previously approved drugs may be a successful avenue for development of pharmaceuticals that are otherwise difficult to craft.

Given their increased stability, ancestral steroid receptors are ideal tools for the extensive mutagenesis required to build robust structure-function relationships for both endogenous and synthetic ligands [13,16]. This same property makes them ideal tools to obtain crystal structures low affinity or weak SR modulators (i.e. lead compounds) that have been recalcitrant to crystallization. However, for ancSR2, given the very high affinity for progesterone and decreased ability for exchange, it may be necessary to express the receptor in the presence of the desired target ligand prior to protein purification and crystallization. 
